# County-Level Socioeconomic Status Adjustment of Acute Myocardial Infarction Mortality Hospital Performance Measure in the U.S.

**DOI:** 10.3390/healthcare9111424

**Published:** 2021-10-22

**Authors:** Sean Daley, Bakthameera Kajendrakumar, Samyuktha Nandhakumar, Christine Personett, Michael Sholes, Swornim Thapa, Chen Xue, Michael Korvink, Laura H. Gunn

**Affiliations:** 1 Department of Public Health Sciences, University of North Carolina at Charlotte, Charlotte, NC 28223, USA; sdaley5@uncc.edu (S.D.); bkajendr@uncc.edu (B.K.); snandhak@uncc.edu (S.N.); cpersone@uncc.edu (C.P.); msholes@uncc.edu (M.S.); sthapa1@uncc.edu (S.T.); cxue1@uncc.edu (C.X.); 2 School of Data Science, University of North Carolina at Charlotte, Charlotte, NC 28223, USA; 3 ITS Data Science, Premier, Inc., Charlotte, NC 28277, USA; michael_korvink@premierinc.com; 4 Faculty of Medicine, School of Public Health, Imperial College London, London W6 8RP, UK

**Keywords:** risk-adjusted, acute myocardial infarction mortality rate, socioeconomic status, hospital performance metric

## Abstract

The U.S. Centers for Medicare and Medicaid Services’ (CMS’s) Hospital Compare (HC) data provides a collection of risk-adjusted hospital performance metrics intended to allow comparison of hospital-provided care. However, CMS does not adjust for socioeconomic status (SES) factors, which have been found to be associated with disparate health outcomes. Associations between county-level SES factors and CMS’s risk-adjusted 30-day acute myocardial infarction (AMI) mortality rates are explored for *n* = 2462 hospitals using a variety of sources for county-level SES information. Upon performing multiple imputation, a stepwise backward elimination model selection approach using Akaike’s information criteria was used to identify the optimal model. The resulting model, comprised of 14 predictors mostly at the county level, provides an additional 8% explanatory power to capture the variability in 30-day risk-standardized AMI mortality rates, which already account for patient-level clinical differences. SES factors may be an important feature for inclusion in future risk-adjustment models, which will have system and policy implications for distributing resources to hospitals, such as reimbursements. It also serves as a stepping stone to identify and address long-standing SES-related inequities.

## 1. Introduction

Social determinants of health consist of the circumstances, settings, and environments in which people are born, develop, play, learn, live, and work [1]. Examples of these determinants include socioeconomic status (SES), education, physical living environment, and access to healthcare. Healthy People 2020 and, more recently, Healthy People 2030 highlight the relevance of social determinants of health with objectives that focus on multiple such determinants, including health care access and quality [2]. Other federal initiatives that aim to improve social determinants of health include the National Partnership for Action to End Health Disparities and the National Prevention and Health Promotion Strategy [3]. Additionally, social determinants of health are addressed in a global setting by the World Health Organization’s Commission on Social Determinants of Health [4]. Organizations, communities, and private institutions can all play a role in addressing inequities stemming from social determinants of health [5,6].

SES factors can inform a patient’s health status and risks [7], as highlighted in the Whitehall Study [8]. This longitudinal study conducted in Great Britain showed that people with low SES experience worse health outcomes and shorter life expectancy. Low SES is also linked to poor housing conditions, fewer recreational opportunities, and increased exposure to crime and violence [9].

Risk adjustment methods, which have become common approaches to measurement of quality of care, aim to provide a fair metric of hospital performance by incorporating information about patient characteristics [10]. Metrics stemming from these methods are used to link hospital performance and financial payouts in pay-for-performance programs, such as the Hospital Value-Based Purchasing (HVBP) program where an acute inpatient prospective payment system (IPPS) payment is tied to measure performance [11]. Addressing social inequities through payment reform is becoming increasingly relevant [12]. However, the Centers for Medicare and Medicaid Services’ (CMS’s) regulatory programs do not yet adjust for SES factors in their risk adjustment mortality models [11].

This manuscript focuses on an acute myocardial infarction (AMI) mortality measure to demonstrate the relevance of SES factors on patient health and hospital performance. Risk-adjusted AMI mortality is a metric used by CMS in the form of a 30-day, risk-standardized mortality measure adjusted by patient characteristics and comorbidities [10]. Multiple SES factors have been identified to be associated with AMI-related mortality. For example, differences by race have been identified, with black patients aged 65–90 experiencing worse AMI-related mortality outcomes than same-aged white patients, and differences are exacerbated in medium or high SES areas [13]. Place of living, coarsely defined as a SES area, neighborhood, or zip code, has also been found to be associated with heterogeneity in patient-level AMI mortality outcomes, with those in lower SES areas associated with worse outcomes [13,14,15,16]. Income, which is directly or indirectly associated with multiple other SES characteristics, has been found to be a major factor associated with other AMI outcome disparities (i.e., hospital readmissions) [15,17,18]. Other factors explored in the literature include exposure to disparate crime rates [19,20], as well as inequities in primary care availability and quality [21], prevalence/affordability of health insurance coverage [22,23], or differences in healthy food options/supermarkets in the area or an inability to afford them [24].

Incorporation of such factors in patient risk adjustment modeling methods is imperative. CMS’s 30-day AMI mortality model does not consider the patients’ SES, even as it can be another determinant to their health outcomes. In this study, we aim to assess whether county-level SES factors are relevant to explain the variability within CMS’s 30-day risk-standardized AMI mortality hospital performance metric in the U.S., and quantify these associations. Incorporation of SES factors into such hospital performance metrics is a necessary step toward increased health equity and improved AMI-related mortality outcomes.

## 2. Materials and Methods

### 2.1. Data

The two primary data sources utilized in this study were the 2017–2018 Area Health Resources Files (AHRF) [25], which are provided by the US Department of Health and Human Services (HHS) Health Resources and Services Administration, and CMS’s Hospital Compare (HC) data [26]. The AHRF dataset provides county-level information on health professions, health facilities, hospital utilization, hospital expenditures, as well as population, socioeconomic, and environmental characteristics [27]. CMS annually compiles HC data (now with the name Hospital Care Compare as of 1 December 2020), which provide information on the quality of care at facilities nationwide [26]. Datasets from the HC program used for this study include hospital general information [28], complications and deaths [29], and unplanned hospital visits [30].

In addition to the aforementioned data, crime data were obtained from the Federal Bureau of Investigation’s (FBI) Uniform Crime Reporting (UCR) Program. The UCR Program provides annual counts of county-level arrests and reported crimes from over 18,000 law enforcement agencies throughout the US [31].

The Institute of Medicine defines safety net hospitals (SNH) as “providers that organize and deliver a significant level of both health care and other health-related services to the uninsured, Medicaid and other vulnerable populations” [32]. Unfortunately, there is no well-defined or widely accepted threshold of what constitutes a significant level of care and services [33]. Thus, we utilized the 303 facilities listed with America’s Essential Hospitals Network, which defines itself as “hospitals provid[ing] a substantial volume of care to low-income patients, the uninsured, and others who face social and economic hardships” [34] as a proxy for SNH status. Due to differences in formatting of strings (i.e., hospital names and locations) across datasets, a fuzzy match algorithm was used to merge data across files [35], and 212 definitive matches of safety net hospitals within the HC dataset were found upon removing false positives, among a total of *n* = 2462 hospitals with available 30-day risk-standardized AMI mortality rates. Safety net hospitals not included in the HC data primarily consisted of children’s hospitals, behavioral health/psychiatric hospitals, and rehabilitation hospitals.

All records in the CMS HC, including datasets concerning complications and deaths in hospitals and unplanned hospital visits with a 30-day risk-standardized AMI mortality rate, were included in the study. This resulted in children’s hospitals and Department of Defense hospitals being excluded.

The outcome variable of interest is the 30-day risk-standardized AMI mortality rate. While the list of possible SES factors that could have been considered is fairly large, a set of relevant SES factors was identified and is listed in Table 1. This list, informed by our literature review, is not comprehensive, but it is meant to provide a proof of concept toward demonstrating the relevance of county-level SES factors to explain hospital outcome variability. Additional factors that may be available could, therefore, further enhance the results provided in this manuscript.

### 2.2. Methods

Income and population density variables were log-transformed due to high skewness, and other count variables were transformed to population rates. Multiple imputation by chained equations (MICE) was used to impute missing data [36]. In order to extract the SES-related variability in 30-day risk-standardized AMI mortality rates, a multivariate linear regression was used (mortality rates were away from the boundaries within a tight range, hence not in violation of linear regression assumptions), and a variable selection approach was performed. For variable pairs with similar definitions and pairwise correlations above 0.95, the older measure was discarded, resulting in the removal of 7 variables prior to the analysis. The Akaike information criterion (AIC) was used during the backward elimination process [37]. We focused on AIC-based model selection since the primary research question is whether county-level SES information is relevant to explain the variability in the 30-day risk-standardized AMI mortality metric, rather than variable selection. The final AIC-based model was defined on the basis of goodness of fit, and, as a secondary outcome of interest, *p*-values from the resulting model were used to assess each SES factor’s significance at α = 0.05. Variance inflation factors (VIFs) were also explored for the final model to assess potential problems regarding multicollinearity in variable/model interpretation.

## 3. Results

Fourteen SES predictors were included upon model selection for the SES-adjusted 30-day risk-standardized AMI mortality analysis. Table 2 contains model summaries after model selection.

The AIC-based optimal model was able to explain 7.5% of the variability in the 30-day risk-standardized AMI-related mortality outcome (*p* < 0.001) with a ratio of sample size to covariates of 188. VIFs were consistently low, with only one value above 5 (% Medicaid Eligible; VIF = 5.52), a majority of VIFs smaller than 2, and a mean VIF of 2.5. Under a commonly used rule-of-thumb of VIF = 10, these values were considered sufficiently low to explore within-model variable significance.

Counties coded as mining-focused (*p* = 0.023; those for which 13% of business earnings or 8% of jobs belong to that sector) and where residents aged 60 and older grew by 15% of more over the 2000–2010 period (retirement destination, *p* = 0.010) were associated with significantly higher 30-day risk-standardized AMI mortality rates. Manufacturing counties showed a non-significant positive association with 30-day risk-standardized AMI mortality rates (*p* = 0.122). Counties with higher per capita personal income (*p* = 0.009) and higher ratios of income to poverty level ≥ 2.00 (*p* = 0.011) were associated with statistically significantly lower 30-day risk-standardized AMI mortality rates. Counties with a larger percentage of its population who are Medicaid-eligible, which is more often associated with younger populations, were significantly negatively associated with 30-day risk-standardized AMI mortality rates (*p* = 0.016), whereas there was a non-significant positive association between dual Medicare/Medicaid eligibility and 30-day risk-standardized AMI mortality rates (*p* = 0.109).

Counties with larger populations of veterans (*p* = 0.002), as well as larger percentages of the population who are Asian (*p* = 0.021), black/African American (*p* = 0.024), and ‘Other’ race (*p* = 0.0709), compared to white populations, were all associated with higher 30-day risk-standardized AMI mortality rates, though the latter group was non-statistically significant. The magnitude was substantially worse for veterans, who appear to be more associated with worse 30-day risk-standardized AMI mortality rates for hospitals in the counties where they reside.

Counties with larger population densities were associated with lower 30-day risk-standardized AMI mortality rates (*p* = 0.033), whereas counties with higher violent crime rates were associated with higher 30-day risk-standardized AMI mortality rates (*p* = 0.012). Finally, designation of the hospital as a safety net facility was non-significantly associated with worse 30-day risk-standardized AMI mortality rates (*p* = 0.127).

## 4. Discussion

In this study, we developed a SES-adjusted model to examine the association between county-level SES factors and 30-day risk-standardized AMI mortality rates. SES factors of county typology and primary economic sector (i.e., retirement and mining), veteran population, race (i.e., Asian and black/African American), and violent crime rate were significant contributing factors to explain higher 30-day risk-standardized AMI mortality rates. Conversely, the SES factors of population density, income, and Medicaid eligibility were associated with lower 30-day risk-standardized AMI mortality rates.

Counties with larger retirement communities were associated with worse 30-day risk-standardized AMI mortality, even as this metric already accounts for age differentials at the patient level. Results from a study that examined data from the longitudinal Health and Retirement Study (HRS) in the U.S. concluded that retirement status was associated with a higher incidence of AMI as compared to those working full-time, controlling for age and sex [38]. Retirement was also found to be associated with higher AMI incidence in a Danish study [39]. Similar findings were noted among those unemployed or with job instability [40]. However, post-AMI relative outcomes (e.g., mortality) were not explored in those studies. In our study, the county-level typology definition of retirement only considered the residents’ age (≥60 years old) and not their actual retirement status. Although age and retirement status are clearly associated, this limitation does not allow for direct mapping of our covariate to retirement status.

Larger proportions of individuals identified with certain races (i.e., African American and Asian) were associated with higher 30-day risk-standardized AMI mortality rates compared to white individuals. This aligns with studies which have shown that African Americans experience worse clinical conditions than whites, including being more likely to be treated by less qualified physicians [41] and treated at hospitals with higher risk adjusted AMI mortality rates after surgery [42].

Population density was found to be associated with better 30-day risk-standardized AMI mortality rates. There could be confounders in this association due to populations with healthier habits living in more densely populated areas, as well as populations with higher income within metropolitan areas when compared to less densely populated rural areas. Violent crimes, which is a proxy for lack of perceived safety, were found to be statistically significantly associated with higher 30-day risk-standardized AMI mortality rates. This could be associated with fewer resources or less qualified physicians willing to work in unsafe areas. It could also relate to the responsiveness of emergency services in unsafe locations, or waiting times at potentially busy emergency rooms. This aligns with hospital safety net designation, which is associated with lower incomes of the populations they serve, and, therefore, such hospitals are also subject to fewer resources. Those hospitals, which may be subject to more stringent budgetary constraints, may also find it difficult to compete for highly qualified personnel when compared to those who serve other populations. However, we found safety net hospital designation to be relevant for inclusion in the final model on the basis of AIC, but non-significantly positively associated with higher 30-day risk-standardized AMI mortality rates. This indicates that safety net designation may be less relevant to 30-day risk-standardized AMI mortality upon accounting for other (potentially correlated) neighborhood-level factors. However, safety net hospital designation had a low pseudo-R-squared (0.035) when regressed against the remaining covariates (but not against the outcome).

Finally, while county-level SES factors were found to be associated with 30-day risk-standardized AMI mortality, patient-level SES factors are likely to provide additional, more granular information to capture the inherent heterogeneity in patient-level SES characteristics. Capturing and accounting for both county-level and patient-level SES factors may further enhance risk-adjustment models.

### Strengths and Limitations

Numerous studies identify socioeconomic factors that relate to AMI-related mortality, but oftentimes focus on a single SES, or focus on a very limited group of those factors [13,14,15,16,18]. Our study aims to explore the joint associations between a relatively large number of county-level SES factors and 30-day risk-standardized AMI mortality. While, for feasibility purposes, we identified a set of potential SES factors, we did so in a sufficiently large number to account for a wide variety of factors, and which was informed by the combined body of knowledge identified in the literature. By performing model selection with a focus on model information content, we conducted a data-driven approach in which the data inform which factors provide the best model to explain the heterogeneity in 30-day risk-standardized AMI mortality rates.

SES factors identified in this study account for about 8% of the total variability in 30-day AMI mortality rates (which already account for patient-level clinical factors), reflecting that there is additional, unaccounted SES-sourced rate heterogeneity that should be incorporated in AMI mortality risk-adjustment models. This is the case despite the study limitation stemming from the different geographical scope of 30-day risk-standardized AMI mortality rates for hospitals (which relate to the populations they serve) and county covariates (which may be substantially different if hospitals serve county populations residing in locations that differ largely from county averages). Some hospitals, especially those located along county boundaries, may serve large populations from neighboring counties. This may more greatly affect hospitals located in smaller, rural locations compared to those located in major metropolitan areas. Furthermore, some patients may prefer to travel to have better access to care in a different hospital than that of their county. Using county-level information may be best for hospitals located in counties with low intra-county variability in SES factors.

Since this study relies on county-level information (and the limitations therein), some additional SES factors, including gender or sexual orientation, were not explored. Such information would not be readily available but should be explored by CMS. However, note that factors such as patient’s age or comorbidities are already included by CMS in the calculation of the 30-day risk-standardized AMI mortality metric. Although individual-level factors such as clinical conditions were already accounted for at the patient level in the current 30-day risk-adjusted AMI mortality rates, the use of patient-level (rather than county-level) SES factors is likely to provide additional granular information to capture the inherent heterogeneity in patient-level SES characteristics.

All data used precede COVID-19, which constitutes a disruptive event to AMI mortality rates. However, all data were not synchronous, due to the heterogeneity of sources. This results in potential mismatches between covariate information across time. However, we do not anticipate this to be highly relevant, since most covariates used in this study are expected to be relatively stable over short periods of time.

When comparing individual-level and county-level SES factors, there may be both overlapping and non-overlapping components, so both approaches accounting for SES are complementary. For example, personal income may be relevant at both the individual and county levels. Two individuals with equal income may experience different AMI-related health outcomes depending on whether they live in poorer or wealthier counties (county-level SES factor), while two individuals with different income may also experience different AMI-related outcomes when living in the same county (patient-level SES factor). Individual income may define personal affordability of resources, while county income characteristics may define their general availability.

## 5. Conclusions

While individual clinical factors are a substantial component of 30-day risk-standardized AMI mortality, SES factors are also associated with heterogeneity of this outcome. County-level SES factors were found to be associated with 30-day risk-standardized AMI mortality, and could be used in conjunction with patient-level SES factors to capture inherent heterogeneity in patient-level health metrics.

While income and wealth redistribution policies (e.g., taxes) and increased social, environmental, and community investments (e.g., education, infrastructure) can palliate some of the SES disparities identified, it may take decades or generations, unfortunately, before such disparities are no longer present or influential on health-related outcomes. Differences in 30-day risk-standardized AMI mortality are, in part, a result of these underlying SES disparities, highlighting that patient clinical characteristics are not the only relevant factors influencing health outcomes. Until relevant policies are implemented and the disparity gap is eliminated or greatly reduced, one immediate change that can be made is the incorporation of SES factors in CMS’s 30-day AMI mortality metric, for example. This remedy, in turn, may help towards bridging existing SES disparity gaps in health outcomes. Accounting for county-level SES factors is an essential improvement to hospital performance metrics, and capturing and accounting for SES factors can further enhance risk-adjustment models. This can result in fairer hospital evaluations, comparisons, and reimbursements via programs such as HVBP, and can act as a stepping stone towards addressing long-standing SES-related inequities.

## Figures and Tables

**Table 1:** List of socioeconomic status (SES) factors selected for the study.

SES Factor	Variable Name (Reference Year(s))
County Typology	Farming Dependent Typology Code (2015)
	Mining Dependent Typology Code (2015)
	Manufacturing Dependent Typology Code (2015)
	Non-Specialized Typology Code (2015)
	Low Education Typology Code (2015)
	Low Employment Typology Code (2015)
	Persistent Poverty Typology Code (2014)
	Population Loss Typology Code (2015)
	Retirement Destination Typology Code (2015)
Per Capita Income	Per Capita Personal Income (2016)
Income	Median Household Income (2016)
Persons/Families Below Poverty Level	% Persons Below Poverty Level (2012–2016)
	% Families Below Poverty Level (2012–2016)
	% Persons in Poverty (2016)
Deep Poverty	% Persons in Deep Poverty (2012–2016)
	% Age ≥ 65 in Deep Poverty (2012–2016)
Ratio of Income to Poverty Level	Ratio of Income to Poverty Level ≥ 2.00 (2012–2016)
Medicaid/Medicare Eligibility	% Medicaid Eligible (2012)
	% Medicare & Medicaid Dual Eligibility (2012)
	% Medicare Eligible (2017)
Health Insurance	% Age ≥ 65, No Health Insurance, & Below 138% of Poverty Level (2016)
	% Age ≥ 65, No Health Insurance, & Below 200% of Poverty Level (2016)
	% Age ≥ 65, No Health Insurance, & Below 400% of Poverty Level (2016)
	% Age 18–64, No Health Insurance, & Below 138% of Poverty Level (2016)
	% Age 18–64, No Health Insurance, & Below 200% of Poverty Level (2016)
	% Age 18–64, No Health Insurance, & Below 400% of Poverty Level (2016)
	% Age ≤ 18, No Health Insurance, & Below 138% of Poverty Level (2016)
	% Age ≤ 18, No Health Insurance, & Below 200% of Poverty Level (2016)
	% Age ≤ 18, No Health Insurance, & Below 400% of Poverty Level (2016)
	% Age ≥ 65, No Health Insurance (2016)
	% Age 18–64, No Health Insurance (2016)
Marketplace Health Insurance Enrollment	% Health Insurance Marketplace Enrollees (2017)
Disability	% Non-institutionalized Disabled Age ≤ 18 (2012–2016)
	% Non-institutionalized Disabled Age 18–64 (2012–2016)
	% Non-institutionalized Disabled Age ≥ 65 (2012–2016)
	% Disabled Enrolled in Medicare (2016)
Food Stamp/SNAP Recipient	% Food Stamp Snap Recipients (2015)
Education	% Aged ≥ 25 with Less than High School Diploma (2012–2016)
	% Aged ≥ 25 with 4 or More Years of College (2012–2016)
Veteran Population	% Veteran Population (2018)
Race	% American Indian or Alaska Native (2010)
	% Asian Population (2010)
	% Black/African American Population (2010)
	% Hispanic/Latino Population (2010)
	% Other Population (2010)
	% White Population (2010)
Core Based Statistical Area (CBSA) Indicator	CBSA Indicator Code (2017)
Rural/Urban	Rural/Urban Continuum Code (2013)
Population Density	Population Density Per Square Mile (2010)
Supplemental Security Income Program Recipients	Households with Supplemental Security Income (2012–2016)
Labor Force	Unemployed in Civil Labor Force (2012–2016)
Incidence Rate of Violent Crimes	FBI Incidence Rate of Violent Crimes (2019)
Hospital Safety Net Designation	Hospital Safety Net Designation (2019)

**Table 2:** Socioeconomic status factors and corresponding coefficient estimates, standard errors, and *p*-values, along with the model’s coefficient of determination, for the resulting model after AIC-based model selection for predicting 30-day risk-standardized acute myocardial infarction (AMI) mortality rates.

SES Variable	Coefficient Estimate	Standard Error	*p-*Value
Intercept	17.782	1.433	<0.001
Mining Dependent Typology Code	0.279	0.123	0.023
Manufacturing Dependent Typology Code	0.138	0.089	0.122
Retirement Destination Typology Code	0.183	0.071	0.010
Per Capita Personal Income	−0.358	0.137	0.009
Ratio of Income to Poverty Level ≥ 2.00	−0.081	0.032	0.011
% Medicaid Eligible	−0.016	0.006	0.016
% Medicare & Medicaid Dual Eligibility	0.047	0.030	0.109
% Veteran Population	0.040	0.013	0.002
% Asian Population	0.013	0.006	0.021
% Black/African American Population	0.006	0.002	0.024
% Other Population	0.012	0.007	0.079
Population Density Per Square Mile	−0.063	0.029	0.033
FBI Incidence Rate of Violent Crimes	0.322	0.128	0.012
Hospital Safety Net Designation	0.139	0.091	0.127
R2 = 0.075; F = 12.46; *p* < 0.001			

## Data Availability

Data are publicly available through the sources referenced in-text.
